# *TNNT2* Missplicing in Skeletal Muscle as a Cardiac Biomarker in Myotonic Dystrophy Type 1 but Not in Myotonic Dystrophy Type 2

**DOI:** 10.3389/fneur.2019.00992

**Published:** 2019-09-27

**Authors:** Francesca Bosè, Laura Valentina Renna, Barbara Fossati, Giovanni Arpa, Valentina Labate, Valentina Milani, Annalisa Botta, Emanuele Micaglio, Giovanni Meola, Rosanna Cardani

**Affiliations:** ^1^Laboratory of Muscle Histopathology and Molecular Biology, IRCCS-Policlinico San Donato, Milan, Italy; ^2^Department of Neurology, IRCCS-Policlinico San Donato, Milan, Italy; ^3^Department of Neurorehabilitation Sciences, Casa Cura Policlinico (CCP), Milan, Italy; ^4^Department of Biomedical Sciences for Health, University of Milan, Milan, Italy; ^5^University Cardiology Unit, IRCCS-Policlinico San Donato, Milan, Italy; ^6^Scientific Directorate, IRCCS Policlinico San Donato, Milan, Italy; ^7^Department of Biomedicine and Prevention, University of Rome “Tor Vergata”, Rome, Italy; ^8^Department of Arrhythmology, IRCCS Policlinico San Donato, Milan, Italy

**Keywords:** myotonic dystrophies, cardiac troponin T, cardiac involvement, skeletal muscle, alternative splicing

## Abstract

Cardiac involvement is one of the most important manifestations of the multisystemic phenotype of patients affected by myotonic dystrophy (DM) and represents the second cause of premature death. Molecular mechanisms responsible for DM cardiac defects are still unclear; however, missplicing of the cardiac isoform of troponin T (*TNNT2*) and of the cardiac sodium channel (*SCN5A*) genes might contribute to the reduced myocardial function and conduction abnormalities seen in DM patients. Since, in DM skeletal muscle, the *TNNT2* gene shows the same aberrant splicing pattern observed in cardiac muscle, the principal aim of this work was to verify if the *TNNT2* aberrant fetal isoform expression could be secondary to myopathic changes or could reflect the DM cardiac phenotype. Analysis of alternative splicing of *TNNT2* and of several genes involved in DM pathology has been performed on muscle biopsies from patients affected by DM type 1 (DM1) or type 2 (DM2) with or without cardiac involvement. Our analysis shows that missplicing of muscle-specific genes is higher in DM1 and DM2 than in regenerating control muscles, indicating that these missplicing could be effectively important in DM skeletal muscle pathology. When considering the *TNNT2* gene, missplicing appears to be more evident in DM1 than in DM2 muscles since, in DM2, the *TNNT2* fetal isoform appears to be less expressed than the adult isoform. This evidence does not seem to be related to less severe muscle histopathological alterations that appear to be similar in DM1 and DM2 muscles. These results seem to indicate that the more severe *TNNT2* missplicing observed in DM1 could not be related only to myopathic changes but could reflect the more severe general phenotype compared to DM2, including cardiac problems that appear to be more severe and frequent in DM1 than in DM2 patients. Moreover, *TNNT2* missplicing significantly correlates with the QRS cardiac parameter in DM1 but not in DM2 patients, indicating that this splicing event has good potential to function as a biomarker of DM1 severity and it should be considered in pharmacological clinical trials to monitor the possible effects of different therapeutic approaches on skeletal muscle tissues.

## Introduction

Myotonic dystrophy type 1 (DM1; OMIM#160900) and type 2 (DM2; OMIM#602668) are dominantly inherited neuromuscular disorders characterized by a multisystemic involvement including muscle weakness, myotonia, respiratory insufficiency, central nervous system impairment, conduction system disease, ventricular dysfunction, and supraventricular and ventricular arrhythmias (1). DM1 is caused by an expanded CTG repeat in the 3′ untranslated region (UTR) of the *Dystrophia Myotonic Protein Kinase* gene (*DMPK*) (2–4), while DM2 is caused by expanded CCTG repeats in intron 1 of the *CCHC-type zinc finger, Nucleic acid Binding Protein* gene (*CNBP*/*ZNF9*) (5). Nuclear accumulation of CUG/CCUG-containing RNA is thought to be toxic, contributing significantly to pathogenesis since the deregulation of several splicing factors, leading to the alteration of alternative splicing of various genes linked to DM symptoms (6). Deregulation of the alternative splicing of the insulin receptor (*INSR*), muscle chloride channel (*CLCN1*), and dystrophin (*DMD*) mRNAs is associated with the insulin resistance (7, 8), myotonia (9–11), and dystrophic process (12), respectively, while missplicing of amphiphysin 2 (*BIN1*), ryanodine receptor 1 (*RYR1*), sarcoplasmic/endoplasmic reticulum Ca^2+^-ATPase SERCA1 (*ATP2A1*), and muscle calcium channel CaV1.1 (*CACNA1S*) may contribute to the skeletal muscle weakness observable in DM1 (13–16).

Cardiac defects affect 80% of individuals with DM1 and represent the second most common cause of death (17, 18). Initially, cardiac involvement manifests as asymptomatic electrocardiographic (ECG) abnormalities, commonly prolongation of the PR interval and QRS duration. In DM2, the frequency and severity of conduction disorders appear to be milder than in DM1 patients; however, in this patient population, a careful cardiac follow-up evaluation is also recommended (19). The most common cardiac manifestations in DM patients include dilated cardiomyopathy ventricular tachycardia and ventricular fibrillation (19–22). Sudden death is considered the result of atrioventricular block or ventricular arrhythmias (23, 24). To date, the molecular mechanisms responsible for cardiac defects in DM are still unclear. However, it has been reported that alteration of the alternative splicing of the cardiac isoform of troponin T (*TNNT2*) and of the cardiac sodium channel (*SCN5A*) might contribute to the reduced myocardial function and conduction abnormalities seen in DM patients (25, 26). *TNNT2* is a gene expressed in embryonic and adult cardiac muscle, and in embryonic skeletal muscle (27). Regulation of alternative splicing of exon 5 leads to exon inclusion in mRNAs produced during early development of heart and skeletal muscle and to exon exclusion in adult heart (28). These two *TNNT2* isoforms confer different calcium sensitivity to the myofilament, affecting the contractile properties of maturing muscle (29, 30). Adult cardiac muscle of DM patients shows alteration of *TNNT2* alternative splicing such that inclusion of exon 5 is inappropriately increased; thus, the expression of this fetal isoform in DM1 patients' heart might contribute to the reduced myocardial function and conduction abnormalities seen in these patients (25).

During skeletal muscle regeneration, newly formed multinucleated myotubes express developmental markers, such as fetal isoforms of MyHC and cardiac-specific markers such as cTnT, which is also expressed in embryonic skeletal muscle (31). It has been reported that *TNNT2* is re-expressed in diseased skeletal muscle from DM patients and from patients with other neuromuscular diseases such as inclusion body myositis (IBM) (32–35). More recently, it has been observed that *TNNT2* is expressed at the mRNA level also in healthy adult skeletal muscle and that, in DM skeletal muscle, an aberrant alternative splicing pattern as in cardiac tissue is observable (25, 34, 36, 37). Thus, our objectives were to identify the biological role of *TNNT2* expression in DM skeletal muscles and to clarify if the expression of the aberrant fetal isoform could be secondary to myopathic changes or if it could reflect the cardiac phenotype of these patients, thus representing a muscular biomarker of cardiac involvement.

## Materials and Methods

The ethical committee Ospedale San Raffaele (Milan, Italy) reviewed and approved this study protocol, which was conducted according to the principles expressed in the Declaration of Helsinki, the institutional regulation, and Italian laws and guidelines. All patients enrolled gave a written informed consent for all blood samples and muscle biopsies used in this study.

### Patients

The study was performed on a total of 24 DM1 and 9 DM2 patients. The diagnosis of DM was based upon the clinical diagnostic criteria set by the International Consortium for Myotonic Dystrophy (38). Fluorescence *in situ* hybridization using a (CAGG)_5_ probe was performed on muscle frozen sections for DM2 diagnosis to verify the presence of nuclear accumulation of mutant RNA (39). DM1 genotyping was performed on genomic DNA obtained from peripheral blood leukocytes according to Valaperta et al. (40). Ten age-matched subjects with no sign of neuromuscular or cardiovascular disease were used as controls (CTR). As internal controls, two patients affected by inclusion body myositis (noDM-IBM) were also included in the study.

### Neurological and Cardiac Evaluation

At the time of muscle biopsy, all DM and control subjects underwent complete clinical neurological and cardiac evaluation. Five-point MRC scale (Medical Research Council) for a total maximum score of 130 was used to evaluate muscle strength. Stage of DM1 disease was determined using the Muscular Impairment Rating Scale (MIRS) (41). Cardiac evaluation included standard 12-lead ECG, 24-h Holter monitoring and 2D-echocardiography. Patients were identified as having cardiac involvement (CI) when presented at least one ECG abnormality (PR interval ≥ 200 ms; QRS duration ≥ 100 ms; QTc > 430 ms in female or >450 ms in male) or left ventricular EF < 50%. Left ventricular hypertrophy (LVH) and dilation (LVD) with rare overt systolic and diastolic dysfunction were also considered since they are relatively frequent findings on echocardiography in DM patients.

### Muscle Biopsies

Biceps brachii (BB) and tibialis anterior (TA) muscle biopsies were taken under sterile conditions from a total of 24 DM1 (12 BB, 12 TA) and 9 DM2 (9 BB) patients enrolled in “The Italian Registry for Myotonic Dystrophy Type 1 and Type 2.” In DM1, both BB and TA muscle biopsies were obtained for research intents, while in DM2 patients, BB biopsies were performed for diagnostic purposes. Muscle biopsies from 10 age-matched healthy subjects (6 BB, 4 TA) were used as CTR. Biopsies of vastus lateralis muscle (VL) were taken from noDM-IBM patients. All patients underwent overnight fasting before blood and muscle sample collection. Muscle tissue was flash frozen in isopentane cooled in liquid nitrogen. Routine histological or histochemical stainings were performed on serial sections (8 μm) for histopathological analysis.

### Immunohistochemistry

Serial sections (6 μm) were air-dried and rehydrated in phosphate buffer solution pH 7.4 (PBS). Sections were incubated with normal goat serum (NGS; Dako, Glostrup, Denmark) at a dilution 1:20 in PBS containing 2% bovine serum albumin (BSA; Sigma-Aldrich, St. Louis, MO, USA) for 20 min at room temperature (RT) to block the non-specific binding sites. Mouse monoclonal primary antibodies against four different myosin heavy chains (MHCs) were applied to sections overnight at 4°C: MHC-fast (MHCf, 1:400 in PBS+2%BSA, Sigma-Aldrich), MHC-slow (MHCs, 1:400 in PBS+2%BSA, Sigma-Aldrich), MHC-neonatal (MHC-neo, 1:10 in PBS+2%BSA, Novocastra), and MHC-embryonal (MHC-emb, 1:20 in PBS+2%BSA, Novocastra). After washing in PBS (3 × 5 min), sections were incubated with goat anti-mouse biotinylated secondary antibody diluted 1:300 in PBS+2%BSA for 1 h. After washing in PBS (3 × 5 min), sections were incubated for 30 min with Vectastain ABC complex (Vector Laboratories, Burlingame, CA, USA) and then with 3,3′-diaminobenzidine (DAB) and hydrogen peroxide for 20 min. Mayer's hematoxylin was used for nuclear counterstain.

### Skeletal Muscle Morphometry and Histopathological Grading

To evaluate skeletal muscle damage, four histopathological parameters characteristic of DM skeletal muscle were taken into account: percentage of centrally nucleated fibers (CNF), number of nuclear clumps, atrophy (AF), and hypertrophy (HF) factors. The percentage of MHC-emb or MHC-neo positive fibers was also taken into account as parameters of muscle regeneration. The percentage of CNF and the number of nuclear clumps were evaluated on hematoxylin and eosin-stained sections. The percentage of CNF was calculated as the number of CNF divided by the total number of fibers in 10 randomly selected fields at a light microscope (magnification 200×). The number of nuclear clumps was evaluated in 10 randomly selected fields at a light microscope (magnification 200×) and the number of nuclear clumps per field was calculated. Measurement of fiber diameter and evaluation of AF and HF were made as previously described by Vihola et al. (42) using Image J (Scion Co.) on images taken on MHCf and MHCs immunostained sections (original magnification 200×). Percentage of embryonal or neonatal positive fibers and positive nuclear clump fibers was evaluated on MHC-neo immunostained sections as number of positive fibers divided by the total number of fibers in 10 randomly selected fields at a light microscope (magnification 200×). Limited to DM and CTR skeletal muscle, a grade of histopathological alteration was evaluated for each biopsy calculating the total score as the sum of the scores for several histopathological parameters. The histopathological score was assigned to each parameter according to the degree of changes observed. For the percentage of CNF: 0 = no CNF; 1 = 0 < %CNF ≤ 10; 2 = 10 < %CNF ≤ 15; 3 = %CNF > 15. For the number of nuclear clumps/field: 0 = no nuclear clumps; 1 = 0 < nuclear clumps ≤ 1; 2 = 1 < nuclear clumps ≤ 3; 3 = nuclear clumps > 3. For AF or HF: 0 = no fiber size variability; 1 = 1 < Factor ≤ 2; 2 = 2 < Factor ≤ 5; 3 = Factor > 5. Due to the low number of MHC-emb positive fibers, only two scores were assigned to this parameter: 0 = no MHC-emb positive fibers; 1 = presence of MHC-emb positive fibers. For the percentage of MHC-neo positive fibers: 0 = no MHC-neo positive fibers; 1 = 0 < %MHC-neo fibers ≤ 1; 2 = 1 < %MHC-neo fibers ≤ 5; 3 = %MHC-neo fibers > 5. Since the most affected muscles showed an evident increase of the connective tissue, for the histopathological score, this parameter was also included: 0 = no increase; 1 = increase.

### RNA Extraction and cDNA Synthesis

Total RNA was extracted from BB and TA biopsies using TRIzol reagent (Invitrogen, Milan, Italy). NanoPhotometer NP80 (Implen) was used to verify RNA quantity and quality. An equal amount of RNA for each sample was retrotranscribed in complementary DNA by the High-Capacity cDNA Reverse Transcription Kit (Applied Biosystems, Monza, Italy) according to the manufacturer's protocol. The resulting cDNAs were used to perform both quantitative real time-PCR and classical PCR.

### Quantitative Real-Time PCR

The expression level of the *TNNT2* gene was measured by quantitative RT-PCR (qRT-PCR) using StepOne Plus Real-Time PCR System (Applied Biosystems) and TaqMan Gene Expression Mastermix. Commercially available TaqMan Gene Expression Assays labeled with FAM dye were used and data were normalized to GAPDH housekeeping gene expression (human TNNT2 Hs00943911_m1; human GAPDH Hs02758991_g1, Applied Biosystems). Each PCR reaction was performed in triplicate and relative gene expression was quantified using the DCt method, normalizing data to the expression of the *GAPDH* gene.

### Alternative Splicing Analysis

PCR for the splicing analysis of the *TNNT2* (cardiac Troponin T), *SERCA1* (Sarcoplasmic/endoplasmic reticulum calcium ATPase 1), *TNNT3* (Troponin T3, Fast Skeletal Type), *DMD* (Dystrophin), and *CLCN1* (skeletal muscle chloride channel voltage-sensitive 1) was performed using Platinum Taq Polymerase (Invitrogen, Carlsbad, CA, USA) according to manufacturer instructions. Classical PCR for splicing analysis for *NFIX* (Nuclear Factor I X), *BIN1* (Amphiphysin 2), *RYR*1 (Ryanodine Receptor 1), *CACNA1S* (Voltage-dependent L-type calcium channel subunit alpha-1S), and *LDB3* (LIM Domani Binding 3) was performed using My Taq Red Mix (Bioline), according to the manufacturer's protocol. For each gene, the primers used for all PCR reactions with their respective temperatures of melting (*T*_m_) and relative spliced exons analyzed are listed in Table 1.

**Table 1:** List of PCR primers used for splicing analysis for each gene with the relative spliced exon.

**Gene**	**Exon**	**Forward primer**	**Reverse primer**	***T*_**m**_ (**°**C)^**a**^**
TNNT2	5	5′-ATAGAAGAGGTGGTGGAAGAGTAC-3′	5′-GTCTCAGCCTCTGCTTCAGCATCC-3′	58
LDB3	11	5′-GACTACCAGGAACGCTTCAACC-3′	5′-GACAGAAGGCCGGATGCTG-3′	62
NFIX	7	5′-GAGCCCTGTTGATGACGTGTTCTA-3′	5′-CTGCACAAACTCCTTCAGTGAGTC-3′	62
SERCA1	22	5′-ATCTTCAAGCTCCGGGCCCT-3′	5′-CAGCTCTGCCTGAAGATGTG-3′	62
TNNT3	F	5′- TTCACCATGTCTGACGAGGAAG-3′	5′- CTTCTGGGATCTTAGGAGCAGTG-3′	50
DMD1	78	5′-TTAGAGGAGGTGATGGAGCA-3′	5′-GATACTAAGGACTCCATCGC-3	58
CACNA1S	29	5′-GCTACTTTGGAGACCCCTGGAA-3′	5′-AGGAGGGTTCGCACTCCTTCTG-3′	60
BIN1	11	5′-AGAACCTCAATGATGTGCTGG-3′	5′-TCGTGGTTGACTCTGATCTCGG-3′	58
RYR1	70	5′-GACAACAAAAGCAAAATGGC-3′	5′-CTTGGTGCGTTCCTGGTCCG-3′	60
CLCN1	7a	5′-GGTTGTCCTGAAGGAATACCTCAC-3′	5′-TCCTCTCCAGTAGTTCCGAACAG-3′	60
GAPDH		5′-AGCCTCCCGCTTCGCTCTCT-3′	5′-GCCAGCATCGCCCCACTTGA-3′	60

a *T_m_, melting temperature*.

Total PCR products were electrophoretically resolved on 2% agarose gel for *CACNA1S, NFIX, LDB3*, and *DMD* genes; metaphor agarose gel for *TNNT2, SERCA1, TNNT3, RYR1*, and *BIN1* genes; and 6% acrylamide gel for the *CLCN1* gene. Qualitative analysis of the amplified products was performed using EtBr-stained gels (Sigma-Aldrich) scanned on a ChemiDoc Universal Hood (Biorad). ImageJ software was used to quantify the intensity of each band and the fraction of abnormally spliced isoform respect to the total amount of isoforms was calculated. The expression level of GAPDH was used as the housekeeping gene. Each PCR was performed on cDNA samples derived from two independent retrotranscriptions.

### Protein Extraction and Western Blot Analysis

To analyze protein expression of cardiac troponin T (cTnT), skeletal muscle biopsies were homogenized in 60 μl of 50 mM Tris–HCl with 5% SDS (pH 7.5) to obtain whole-cell protein extracts. Samples were incubated on ice for 15 min and then centrifuged at 5,700 × *g* for 15 min at 4°C, and the supernatant was collected and stored at −80°C. As positive control, whole protein extract from human heart auricula was also obtained.

Protein concentration was determined using Pierce BCA Assay Kit (Thermo Scientific, Rockford, USA) and 25 μg of proteins was separated by SDS-PAGE and transferred to nitrocellulose membranes. Non-specific binding was blocked with Tris–HCl buffer, pH 7.5 (TBS), containing 5% BSA and then membranes were incubated with primary antibodies: mouse monoclonal anti-cTnT (clone 1C11, Abcam, 1:2,000) and mouse monoclonal anti-αTubulin (clone B-5-1-2, Sigma-Aldrich, 1:1,000) used as an internal loading control. After washing with TBS + 0.3% Tween20, membranes were incubated with HRP-conjugated anti-mouse antibody (Jackson ImmunoResearch Laboratories, Inc.) diluted 1:5,000 in TBS + 5%BSA + 0.2% Tween 20. Super Signal West Pico Chemiluminescent Substrate (Thermo Scientific, Meridian Rd., Rockford, USA) was used for immunodetection.

### Statistical Analysis

Categorical variables are presented as proportions, and continuous variables are presented as mean (± SD) and median [interquartile range].

For evaluation of differences in qRT-PCR and alternative splicing analysis between CTR and DM1 or DM2 patients, nonparametric, Kruskal–Wallis test was used. Dwass, Steel, Critchlow–Fligner multiple comparison procedure was used to compare levels where Kruskal–Wallis test was statistically significant. Correlation analysis was performed using nonparametric Spearman Rho. All *p*-values are two-tailed and considered significant if *p* < 0.05. Statistical analysis was performed using SAS software, version 9.4 (SAS Institute, Inc., Cary, NC).

## Results

### Patients

This study was performed on a total of 24 DM1 and 9 DM2 patients compared to 12 control subjects. The DM1 cohort was represented by patients affected by the mild form (range of CTG repeat expansion E1 = 50–149) or by the classical adult form of the disease (range of CTG repeat expansion E2 = 150–1,000); the DM2 cohort was represented by patients with classical Proximal Myotonic Myopathy (PROMM) phenotype. DM patients were divided into two subgroups based on the presence (CI) or not (NCI) of cardiac involvement. Among DM1, 10 patients were classified as NCI while 14 were classified as CI. Among DM2, five patients were classified as NCI while four were classified as CI. At echocardiogram, LVH was observed in two DM patients (DM1-7 CI and DM2-9 CI) who also were pacemaker carriers. No patients presented LVD and systolic or diastolic dysfunctions except for DM1-7 CI who showed diffuse left ventricular hypokinesis. All patients were ambulant and mildly–moderately affected (Table 2). As internal controls, two patients affected by inclusion body myositis (noDM-IBM) were used. Clinical data and skeletal muscle biopsy were collected during patient hospitalization. Clinical data on DM and control patients are reported in Table 2.

**Table 2:** Clinical data on control, DM1, and DM2 patients.

	**Gender**	**Age at biopsy**	**Age at onset**	**CTG repeat size**	**MRC^**a**^**	**MIRS^**b**^**	**PR interval^**c**^**	**QRS duration^**d**^**	**QTc^**e**^**	**%EF^**f**^**	**Drugs^**g**^**
CTR1 (BB)	F	42	–	–	–	–	168	90	403	66	None
CTR2 (BB)	F	50	–	–	–	–	157	84	410	68	None
CTR3 (BB)	F	44	–	–	–	–	184	79	415	63	None
CTR4 (BB)	F	52	–	–	–	–	173	82	404	65	None
CTR5 (BB)	M	44	–	–	–	–	165	77	412	65	None
CTR6 (BB)	F	47	–	–	–	–	176	92	417	69	Levothyroxine
CTR7 (TA)	M	26	–	–	–	–	168	94	404	65	None
CTR8 (TA)	F	29	–	–	–	–	159	92	410	68	None
CTR9 (TA)	M	24	–	–	–	–	165	84	408	62	None
CTR10 (TA)	F	27	–	–	–	–	172	79	412	65	None
DM1-1 NCI (BB)	F	45	35	460	103	4	160	95	420	72	Levothyroxine
DM1-2 NCI (BB)	M	66	50	100	130	1	184	88	396	68	None
DM1-3 NCI (BB)	M	42	36	390	128	2	172	82	428	64	None
DM1-4 NCI (BB)	F	48	30	240	105	4	168	82	422	79	None
DM1-5 CI (BB)	M	42	10	950	105	4	202	124	429	58	None
DM1-6 CI (BB)	F	65	15	300	117	3	156	204	520	65	ASA, Acarbose, Levothyroxine, Metformin
DM1-7 CI (BB)	M	43	18	500	119	3	Pacemaker carrier				Enalapril, ASA
DM1-8 CI (BB)	M	43	33	240	111	3	214	126	403	66	None
DM1-9 CI (BB)	F	45	40	300	127	3	216	116	440	68	Flecainide
DM1-10 CI (BB)	M	50	28	220	113	4	202	110	425	65	None
DM1-11 CI (BB)	M	39	37	890	118	3	210	107	434	63	None
DM1-12 CI (BB)	F	56	31	490	118	3	218	112	412	65	Levothyroxine, Enalapril, ASA
DM2-1 NCI (BB)	F	50	39	–	125	–	124	86	422	64	None
DM2-2 NCI (BB)	M	61	55	–	118	–	182	82	405	72	ASA, Metformin
DM2-3 NCI (BB)	M	59	65	–	124	–	160	72	386	66	ASA, Levothyroxine, Metformin
DM2-4 NCI (BB)	F	51	45	–	129	–	164	79	425	62	None
DM2-5 NCI (BB)	F	56	31	–	123	–	156	87	423	60	ASA, Metformin, Insulin, Simvastatin
DM2-6 CI (BB)	F	48	53	–	124	–	220	70	424	60	None
DM2-7 CI (BB)	M	64	57	–	129	–	226	114	417	71	ASA
DM2-8 CI (BB)	M	56	50	–	126	–	164	110	432	66	Warfarin
DM2-9 CI (BB)	M	61	27	–	118	–	Pacemaker carrier				Mexiletine, Simvastatin
DM1-13 NCI (TA)	F	31	31	560	128	1	152	80	379	67	None
DM1-14 NCI (TA)	F	29	18	490	126	2	144	84	402	56	Mexiletine
DM1-15 NCI (TA)	F	34	Unknown	255	126	3	174	86	402	64	None
DM1-16 NCI (TA)	F	22	20	>800	129	2	142	96	420	72	None
DM1-17 NCI (TA)	M	29	14	>800	129	3	167	89	411	65	None
DM1-18 NCI (TA)	M	35	14	680	112	3	194	99	417	68	Mexiletine
DM1-19 CI (TA)	F	44	35	360	125	3	248	109	409	56	Levothyroxine
DM1-20 CI (TA)	F	22	2	455	113	4	232	109	419	62	None
DM1-21 CI (TA)	F	42	16	290	119	3	262	142	446	65	None
DM1-22 CI (TA)	F	39	33	>800	125	4	202	130	420	54	Mexiletine
DM1-23 CI (TA)	F	30	11	>800	114	3	168	164	460	64	Modafinil
DM1-24 CI (TA)	M	33	21	220	130	2	216	107	436	60	Mexiletine
noDM-IBM1 (VL)	F	68	–	–	122		153	86	402	65	Atenolol
noDM-IBM2 (VL)	F	63	–	–	130		165	82	402	66	Metformin

*BB, biceps brachii; TA, tibialis anterior; VL, vastus lateralis; F, female; M, male.*

a *Medical Research Council, scale for muscle strength; scale (0–5 grade) on 13 muscles at both sides in the upper and lower limbs for a total of 130 maximum score.*

b *Muscle Impairment Rating Scale, stage of the disease for myotonic dystrophy type 1 (DM1) patients (41).*

c *PR interval normal value <200 ms.*

d *QRS duration normal value <100 ms.*

e *QTc normal values ≤430 ms in female or ≤450 ms in male.*

f *Left ventricular ejection fraction normal value >50%.*

g *Therapeutic treatment at the time of muscle biopsy.*

### Muscle Histopathology and Immunohistochemistry

**Figure 1:**
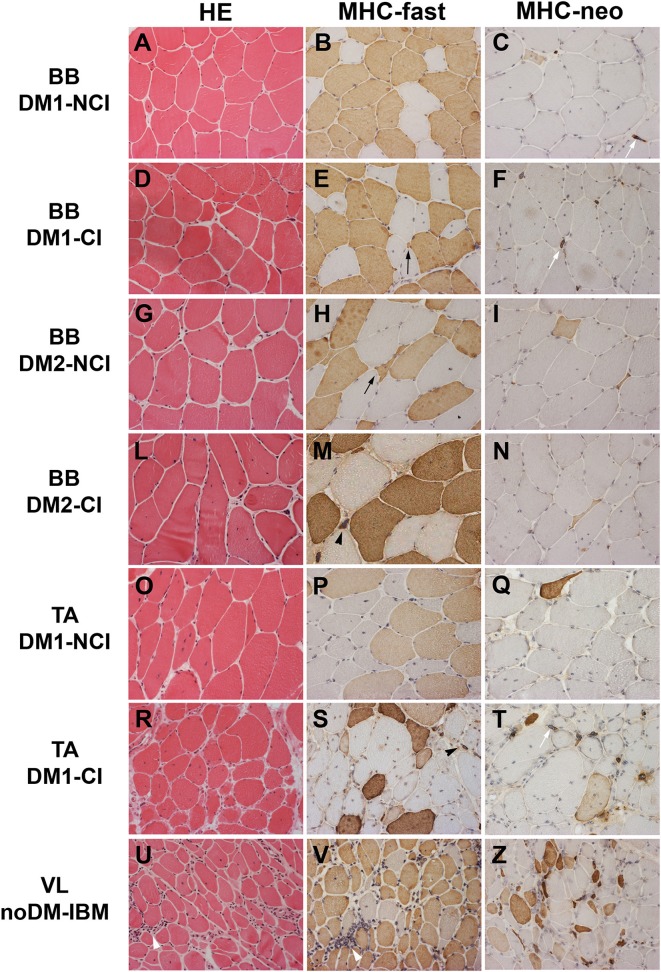
Hematoxylin and eosin (HE) staining and immunostaining of fast myosin heavy chain (MHC-fast) and neonatal myosin heavy chain (MHC-neo) of representative DM1 **(A–F)** and DM2 **(G–N)** biceps brachii (BB), DM1 tibialis anterior (TA) **(O–T)**, and noDM-IBM **(U–Z)** vastus lateralis (VL). DM1 and DM2 patient groups have been divided into two subgroups according to the presence of cardiac abnormalities: NCI, no cardiac involvement; CI, cardiac involvement. Patient group and investigated proteins are indicated. Both DM1 and DM2 show a high fiber size variability **(A,D,G,L,O,R)**. In DM1 atrophic fibers (black arrow) and nuclear clumps (black arrowhead) are both fast (brown fibers) or slow fibers (unstained fibers) **(B,E,P,S)** while in DM2, essentially all atrophic fibers (black arrow) and nuclear clumps (black arrowhead) are identified as fast fibers (brown fibers; **H,M**). Only few atrophic fibers (brown fibers) and almost all nuclear clumps (white arrow) express MyHC-neo **(C,F,I,N,Q,T)**. In the noDM-IBM patient, skeletal muscle shows the presence of numerous atrophic fibers, both fast and slow myosin positive, and several inflammatory infiltrates (white arrowhead) **(U,V)**. Numerous MHC-neo positive fibers are present **(Z)**. Original magnification 200×.

The results of the quantitative analysis of histopathological parameters of routine stained sections and of immunostained sections are reported in Table 3. Histological and immunohistochemical stainings revealed no myopathic changes in skeletal muscle biopsies from CTR. Routine stainings performed on muscle BB and TA transverse sections revealed the presence of the histological alterations commonly observable in DM skeletal muscle such as nuclear clump fibers, nuclear centralization, fiber size variability, and fibrosis (Figure 1). When considering the histopathological analysis, in BB muscle biopsies, a clear increase in percentage of CNF and in the number of nuclear clumps was present in both DM1 and DM2 patients compared to CTR. The percentage of CNF was significantly higher in DM1-CI and in DM2-CI compared to CTR muscles, whereas the number of nuclear clumps appeared to be significantly higher in DM1-CI and in DM2-NCI compared to CTR muscles. A high number of nuclear clumps was evident also in DM2-CI even if not significantly different from CTR due to the high interindividual variability. In TA muscles, the percentage of CNF was significantly higher in DM1-CI compared to CTR. No significant differences in the number of nuclear clumps were found between DM1-CI and DM1-NCI compared to CTR. The immunohistochemical staining of MHC-slow and MHC-fast myosin allowed better evaluation of type 1 and type 2 fiber atrophy (AF) and hypertrophy (HF) factors. In DM1 BB, the AF was significantly higher in DM1-CI compared to CTR, while no differences were found in HF. No significant differences were found in AF and HF between DM1 TA or DM2 and the corresponding CTR groups. The immunostaining of two fetal isoforms of myosin showed that in DM1 and DM2 patients, only very few atrophic fibers in the most severely affected muscles expressed MHC-emb and no nuclear clumps were positive for this myosin isoform. Indeed, no significant differences were observed in the percentage of MHC-emb fibers in both DM1 (BB and TA) and DM2 muscles compared to CTR. The expression of MHC-neo was present in highly atrophic fibers and in almost all nuclear clumps of DM1 and DM2 muscle. In BB muscles, the percentage of MHC-neo fibers appear to be significantly higher in DM2-NCI compared to CTR, while in TA muscles, this parameter was significantly higher in DM1-CI compared to CTR. To evaluate the global degree of myopathic changes observed by microscopic analysis, a histopathological score was assigned to each DM biopsy. The results of this evaluation showed that among the BB muscles, the histopathological score was higher in both DM1 and DM2 with significant differences in DM1-CI and in the two DM2 subgroups compared to CTR. In TA, both DM1 subgroups showed a score higher than that observed in CTR, but reaching statistical significance only in DM1-CI. As expected, in DM1 patients, the histopathological score appeared to be higher in TA than in BB muscles even if with a difference that is not statistically significant. No statistically significant differences were found between the CI and NCI subgroups in both DM1 and DM2 muscles in any of the parameters considered. In noDM-IBM patients, routine staining showed a fiber size variability due to the presence of atrophic fibers and endomysial inflammatory infiltrations (Figures 1U–Z). The immunohistochemical staining revealed an increase of the atrophy factor and of the percentage of MHC-emb fibers compared to CTR muscles. Moreover, a clear increase in the percentage of MHC-neo fibers was also observed compared to CTR and DM skeletal muscles.

**Table 3:** Quantitative analysis of histopathological parameters and muscle pathology score.

	**CTR** **(BB = 6)^**a**^**	**DM1-NCI** **(BB = 4)**	**DM1-CI** **(BB = 8)**	**KW** ***p***	**DM2-NCI** **(BB = 5)**	**DM2-CI** **(BB = 4)**	**KW** ***p***	**CTR** **(TA = 4)**	**DM1-NCI** **(TA = 6)**	**DM1-CI** **(TA = 6)**	**KW** ***p***	**noDM-IBM** **(VL = 2)**
% CNF	1.21 ± 1.66 0.75 [0.00–1.27]	7.59 ± 10.39 4.02 [0.28–14.91]	19.47 ± 18.75^**^ 12.55 [9.43–21.04]	0.014	10.61 ± 7.10 9.73 [9.02–13.08]	26.42 ± 7.73^*^ 25.25 [20.01–32.82]	0.008	± 0.00 0.00 [0.00–0.00]	18.47 ± 14.73 18.84 [6.43–28.83]	50.03 ± 39.35^*^ 41.18 [26.30–88.30]	0.016	0.87 ± 0.87
Nuclear clumps/field	± 0.00 0.00 [0.00–0.00]	0.50 ± 0.59 0.35 [0.05–0.95]	1.55 ± 1.15^**^ 1.25 [0.85–2.05]	0.004	1.30 ± 0.72^**^ 1.00 [0.90–1.90]	1.55 ± 2.23^**^ 0.45 [0.40–2.70]	0.007	± 0.00 0.00 [0.00–0.00]	0.61 ± 0.90 0.10 [0.00–1.33]	1.25 ± 2.63 0.24 [0.00–0.43]	0.301	0.00 ± 0.00
AF	0.83 ± 0.27 0.83 [0.67–0.94]	3.17 ± 3.78 1.56 [1.06–5.28]	3.52 ± 2.10 3.07 [2.47–5.11]	0.055	3.00 ± 2.47 1.97 [1.57–4.06]	1.96 ± 1.53 1.45 [0.91–3.01]	0.143	0.29 ± 0.09 0.31 [0.22–0.37]	2.72 ± 3.32 1.38 [0.18–5.06]	2.86 ± 2.16 2.75 [1.17–5.07]	0.289	5.44 ± 1.93
HF	0.68 ± 0.23 0.64 [0.59–0.85]	1.79 ± 2.54 0.74 [0.25–3.34]	0.77 ± 0.50 0.65 [0.35–1.22]	0.989	1.08 ± 1.01 0.79 [0.36–1.46]	2.38 ± 0.93 2.16 [1.68–3.09]	0.072	2.01 ± 1.05 1.91 [1.12–2.90]	1.80 ± 1.60 1.55 [0.56–2.05]	± 4.51 6.37 [1.36–7.34]	0.147	0.28 ± 0.28
% MHC-emb fibers	0.08 ± 0.19 0.00 [0.00–0.00]	± 0.00 0.00 [0.00–0.00]	0.27 ± 0.31 0.19 [0.00–0.50]	0.173	0.57 ± 0.55 0.70 [0.00–0.73]	± 0.00 0.00 [0.00–0.00]	0.100	± 0.00 0.00 [0.00–0.00]	0.89 ± 1.13 0.54 [0.00–1.52]	1.39 ± 1.07 1.19 [0.69–2.52]	0.082	7.62 ± 4.81
% MHC-neo fibers	0.44 ± 0.66 0.00 [0.00–0.72]	0.43 ± 0.61 0.21 [0.00–0.85]	2.20 ± 2.30 1.43 [0.71–3.25]	0.086	3.56 ± 2.69^*^ 2.43 [2.01–3.61]	1.40 ± 0.91 1.07 [0.80–2.01]	0.012	± 0.00 0.00 [0.00–0.00]	2.79 ± 2.68 2.16 [0.00–5.56]	5.65 ± 5.57^*^ 4.04 [1.11–8.86]	0.027	40.07 ± 4.05
Muscle pathology score	± 0.89 1.00 [0.00–2.00]	5.55 ± 3.11 6.50 [3.50–7.50]	8.12 ± 2.47^**^ 9.00 [7.50–9.00]	0.003	7.20 ± 1.64^*^ 7.00 [6.00–7.00]	8.50 ± 1.19^*^ 7.50 [7.00–10.00]	0.004	1.50 ± 0.58 1.50 [1.00–2.00]	7.50 ± 4.76^*^ 7.50 [3.00–12.00]	11.00 ± 2.83^*^ 12.00 [8.00–13.00]	0.015	/

*Data are mean (± SD)/median [Q1–Q3].*

a *Number of muscles analyzed.*

*%CNF, percent of central nuclear fiber; AF, atrophy factor; HF, hypertrophy factor; KW, Kruskal–Wallis test; BB, biceps brachii: TA, tibialis anterior; VL, vastus lateralis.*

* *p < 0.05,*

** *p < 0.01 Dwass, Steel, Critchlow–Fligner multiple post-hoc comparison procedure: patient group vs. CTR group*.

### *TNNT2* Expression and Alternative Splicing

**Figure 2:**
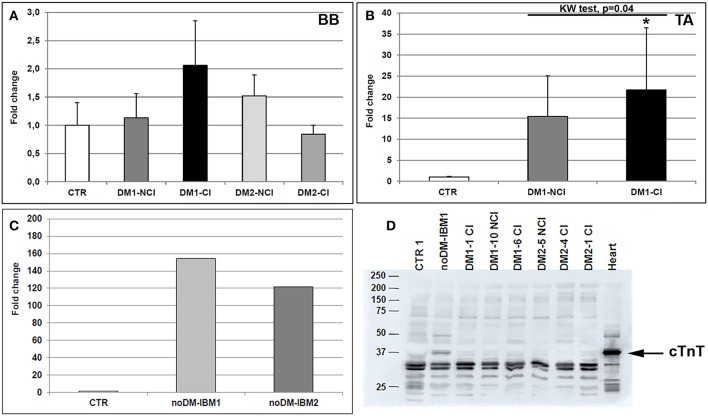
**(A–D)** mRNA and protein *TNNT2* expression in skeletal muscle. *TNNT2* gene expression measured by qRT-PCR in biceps brachii (BB) muscle samples **(A)** and tibialis anterior (TA) muscle samples **(B)** from CTR (BB = 6; TA = 4), DM1 (BB = 12; TA = 12), and DM2 (BB = 9) patients. DM1 and DM2 patient groups have been divided into two subgroups according to the presence of cardiac abnormalities: NCI, no cardiac involvement; CI, cardiac involvement. The expression level of *TNNT2* has been quantified in skeletal muscle (vastus lateralis) of patients affected by inclusion body myositis (noDM-IBM) **(C)**. Histograms represent mean values and bars represent standard error of the mean (SEM). *GAPDH* has been chosen as the housekeeping, internal control. Each PCR reaction has been performed in triplicate. The differences between subgroups have been assessed by nonparametric, Kruskal-Wallis test. Results from Dwass, Steel, Critchlow**–**Fligner multiple *post-hoc* comparison procedure: NCI or CI subgroups vs. CTR, *p < 0.05. **(D)** Representative western blot analysis of cTnT protein expression (37 kDa) in muscle biopsies obtained from CTR, noDM-IBM, DM1, and DM2 patients. Whole protein extract from human heart auricula has been used as positive control.

Total *TNNT2* mRNA expression was analyzed in both control and DM muscle biopsies by qRT-PCR (Figures 2A–C). *TNNT2* expressed both in BB and in TA obtained from CTR patients. When considering BB muscles, a slight increase in mRNA expression was present in DM1 and DM2 patients even if it is not significantly different from CTR. The higher levels of *TNNT2* were observed in DM1-CI but without statistical differences compared to CTR BB. In DM1-NCI and DM2 patients, *TNNT2* expression appeared to be similar to that observed in CTR BB muscles (Figure 2A). In TA muscles, *TNNT2* expression was statistically higher in DM1 compared to CTR TA (Mann–Whitney, *p* = 0.01). When considering the two subgroups, *TNNT2* was higher in both DM1-NCI and DM1-CI compared to CTR subjects but with a statistically significant difference only for DM1-CI (Figure 2B). In noDM-IBM, a more than 100-fold change increase in *TNNT2* expression compared to CTR BB was found (Figure 2C). In order to verify if *TNNT2* mRNA was translated into protein in adult healthy and diseased skeletal muscle, cTnT protein expression was analyzed by western blot. The primary antibody detected the typical band at the molecular weight of cTnT (37 kDa) in cardiac tissue. This band was present also in diseased skeletal muscle of noDM-IBM patients but absent both in CTR healthy muscle and in diseased muscle of DM1 and DM2 patients (Figure 2D). The cTnT antibody detected additional bands in all samples at molecular weights below that of cTnT; however, these bands could represent Slow or Fast skeletal TnT as identified using LC-MS/MS by Schmid et al. (43). Since our study did not focus on cTnT protein expression, we thus cannot be sure in all instances that the mRNA signals resulted in altered protein expression.

**Figure 3:**
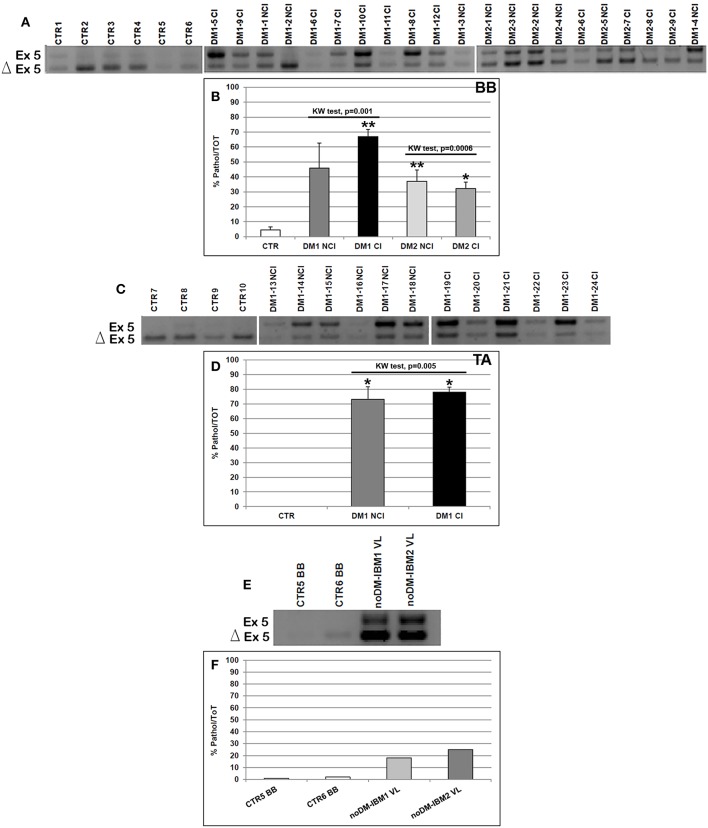
**(A–F)** *TNNT2* alternative splicing in skeletal muscle. **(A,C,E)** Panels showing the reverse transcriptase-polymerase chain reaction (RT-PCR) splicing assay of the *TNNT2* gene in biceps brachii (BB) **(A)** and tibialis anterior (TA) muscle samples **(C)** from CTR, DM1, and DM2 patients. DM1 and DM2 patient groups have been divided into two subgroups according to the presence of cardiac abnormalities: NCI, no cardiac involvement; CI, cardiac involvement. *TNNT2* alternative splicing has been analyzed in vastus lateralis of patients affected by inclusion body myositis (noDM-IBM) **(E)**. The products that include the specific exon 5 (Ex) and those that exclude the specific exon 5 (Δ) have been indicated. **(B,D,F)** Analysis of the percentage of altered isoform (exon 5 including) expression of the *TNNT2* gene in BB **(B)**, TA **(D)**, and in noDM-IBM muscle samples **(F)**. Histograms represent mean values and bars represent SEM. *GAPDH* has been chosen as the housekeeping, internal control. Each PCR reaction has been performed in triplicate. The differences between subgroups have been assessed by nonparametric, Kruskal**–**Wallis test. Results from Dwass, Steel, Critchlow**–**Fligner multiple *post-hoc* comparison procedure: NCI or CI subgroups vs. CTR, *p < 0.05, **p < 0.01.

As expected, the analysis of the alternative splicing of the *TNNT2* gene showed that the fetal isoform (exon 5 including) was significantly more expressed in DM1 BB (Mann–Whitney: *p* = 0.001), DM2 BB (Mann–Whitney: *p* = 0.004), and DM1 TA (Mann–Whitney: *p* = 0.001) compared to the corresponding CTR muscles. When considering the BB and TA subgroups, both CI and NCI showed a significantly higher expression of *TNNT2* fetal isoform compared to CTR except for DM1-NCI BB, which showed a high interindividual variability (Figures 3A–D). However, among all muscles analyzed, the *TNNT2* missplicing is more evident in DM1 (both BB and TA) than in DM2 muscles (Figures 3A,C). Indeed, fetal isoform is more expressed (more than 50% of the total) than the adult isoform in all DM1 BB and TA muscles except for two patients (DM1-2 NCI and DM1-16 NCI) who did not present cardiac abnormalities, and their muscle tissue did not show any histopathological alterations. On the contrary, almost all DM2 muscles expressed more adult than fetal isoform (Figure 3A). Among internal controls, the fetal isoform expression was slightly higher in noDM-IBM patients compared to CTR; however, the amount of fetal isoform was clearly lower than the adult one (Figures 3E,F).

### Skeletal Muscle-Specific Genes Alternative Splicing

**Figure 4:**
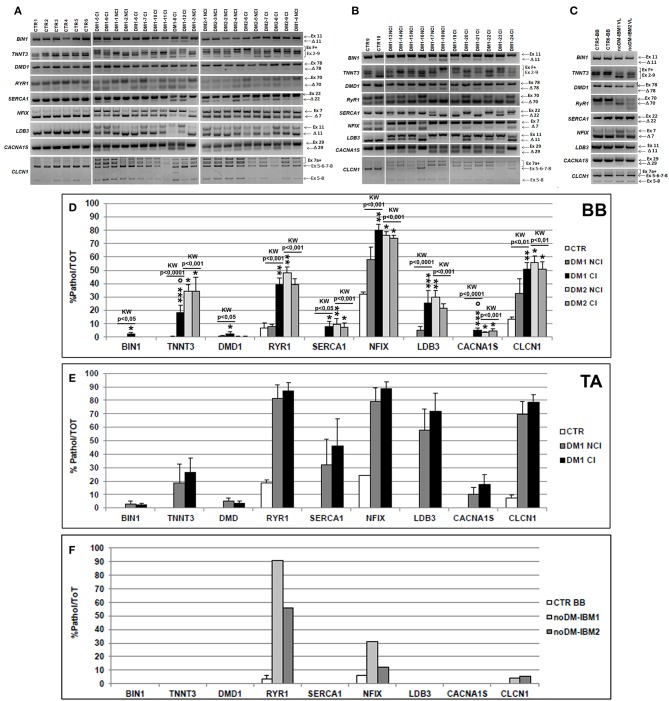
**(A–C)** Alternative splicing of muscle-specific genes in skeletal muscle. Panels showing the reverse transcriptase-polymerase chain reaction (RT-PCR) splicing assay of *BIN1, TNNT3, DMD, RYR1, SERCA1, NFIX, LDB3, CACNA1S*, and *CLCN1* genes in biceps brachii (BB) **(A)** and tibialis anterior (TA) muscle samples **(B)** from CTR, DM1, and DM2 patients. DM1 and DM2 patient groups have been divided into two subgroups according to the presence of cardiac abnormalities: NCI, no cardiac involvement; CI, cardiac involvement. Alternative splicing of the muscle-specific genes has been analyzed in vastus lateralis of patients affected by inclusion body myositis (noDM-IBM) **(C)**. The products that include the specific (Ex) and those that exclude the specific exon (Δ) have been indicated. Analysis of the percentage of altered isoforms expression in BB **(D)**, TA **(E)**, and in noDM-IBM muscle samples **(F)**. Histograms represent mean values and bars represent SEM. *GAPDH* has been chosen as the housekeeping, internal control. Each PCR reaction has been performed in triplicate. The differences between subgroups have been assessed by nonparametric, Kruskal–Wallis test. Results from Dwass, Steel, Critchlow–Fligner multiple *post-hoc* comparison procedure: NCI or CI subgroups vs. CTR, *p < 0.05, **p < 0.01; NCI vs. CI, °*p* < 0.05.

We have analyzed the splicing isoforms of several muscle-specific genes (*BIN1, TNNT3, DMD, RYR1, SERCA1, NFIX, LDB3, CACNA1S*, and *CLCN1*) that are linked to impaired muscle functions in DM pathologies. In BB muscles, except for *BIN1* (in both DM1 and DM2 patients), *DMD1* (in DM2 patients), and *SERCA1* (in DM1 patients), a statistically significant alteration of alternative splicing of the genes examined was present in DM1 and DM2 compared to CTR muscles (data not shown). The differential abnormal splicing patterns of *TNNT3, RyR1, SERCA1, LDB3*, and *CLCN1* appeared more pronounced in DM2 BB than in DM1 BB. When considering DM1 BB, the alterations of the alternative splicing of the genes considered were more evident in DM1-CI with *TNNT3* and *CACNA1S* showing abnormal splicing with significant differences between DM1-NCI and DM1-CI (Figures 4A,D). In DM2 BB, the splicing pattern was similar in DM2-NCI and DM2-CI (Figures 4A,D). In TA muscles, the splicing alterations appeared to be higher in both DM1-NCI and DM1-CI compared to CTR; however, statistical analysis of the differences between DM1 and CTR groups could not be performed since only two CTR subjects were analyzed. Considering the two subgroups, the splicing alterations were slightly higher in DM1-CI but with no significant differences compared to DM1-NCI (Figures 4B,E). Despite the important histopathological alterations, noDM-IBM patients showed a clearly higher expression of the fetal than adult isoform except for the *RYR*1 gene (Figures 4C,F).

### Correlations

The total mRNA expression level of *TNNT2* was correlated with the percentage of fibers expressing MHC-neo isoform in order to verify if a regenerative process was present in the muscles examined. The correlation with the percentage of MHC-emb fibers was not considered since the number of positive fibers resulted very low in DM skeletal muscles. Total expression significantly correlated with the percentage of MHC-neo positive fibers when considering both all muscle samples examined (Spearman test: *r* = 0.45, *p* = 0.002) and also when considering only the DM muscles (Spearman test: *r* = 0.52, *p* = 0.0003). Among BB muscles, no correlation was evident in both DM1 and DM2 patients, except for DM1-CI patients (Spearman test: *r* = 0.57, *p* = 0.03). In DM1 TA muscles, the *TNNT2* expression significantly correlated with the percentage of MHC-neo positive fibers either when considering all TA muscles (Spearman test: *r* = 0.82, *p* = 0.0002) or when considering DM1-NCI (Spearman test: *r* = 0.64, *p* = 0.05) and DM1-CI (Spearman *r* = 0.84, *p* = 0.004) separately. In order to verify if the expression levels of *TNNT2* were related to DM histopathological alterations, the correlation between total mRNA expression and DM histopathological score was performed. A clear correlation was found when considering all the DM muscles examined in this study (Spearman test: *r* = 0.54, *p* = 0.0002). No correlation was present when considering DM1 BB and DM2 BB muscles separately, while a significant correlation was evident in TA muscle of DM1 patients (Spearman test: *r* = 0.83, *p* = 0.0001) and in both DM1-NCI (Spearman test: *r* = 0.77, *p* = 0.01) and DM1-CI patients (Spearman test: *r* = 0.82, *p* = 0.006).

Results regarding correlations between the total expression of *TNNT2* and patient clinical data are reported in Supplementary Table 1. When considering all the muscles analyzed, no correlations were found between the total expression of *TNNT2* and patient age, while a significant correlation was present in DM2 and in DM1 TA. Regarding DM1 patients, cTnT expression did not correlate with age at onset, CTG repeat expansion size, or MIRS. A significant correlation was found between cTnT expression and MRC in the DM1 TA group.

Since several studies on muscular dystrophies reported that splicing changes may be a general phenomenon of muscle disease and can be secondary to muscle regeneration (44, 45), splicing alteration of *TNNT2* and of the muscle-specific genes analyzed was correlated to the percentage of MHC-neo fibers and to DM histopathological score. Regarding the *TNNT2* gene, splicing alteration significantly correlates with the percentage of MHC-neo fibers either when considering all the muscles analyzed (Spearman test: *r* = 0.45, *p* = 0.002) and all the DM muscles (Spearman test: *r* = 0.52, *p* = 0.0003). Among BB muscles, this correlation resulted significant in DM1 patients (Spearman test: *r* = 0.56, *p* = 0.01) and in the subgroup of DM1-CI patients (Spearman test: *r* = 0.66, *p* = 0.01), as well as in DM2 patients (Spearman test: *r* = 0.57, *p* = 0.03) and in the subgroup of DM2-NCI patients (Spearman test: *r* = 0.74, *p* = 0.01). This correlation resulted significant also in DM1 TA (Spearman test: *r* = 0.58, *p* = 0.02) and in DM1-CI TA muscles (Spearman test: *r* = 0.82, *p* = 0.007). A clear correlation was also present between the expression of the fetal *TNNT2* isoform and DM histopathological score either when considering all DM muscles (Spearman test: *r* = 0.68, *p* < 0.0001) and DM1 BB (Spearman test: *r* = 0.80, *p* < 0.0001), DM2 BB (Spearman test: *r* = 0.67, *p* = 0.008), and DM1 TA (Spearman test: *r* = 0.61, *p* = 0.01) muscles separately. Similar results were obtained when considering the correlation between the percentage of MHC-neo fibers or DM histopathological score and the splicing alteration of muscle-specific genes considered in this study (Supplementary Tables 2, 3).

Results regarding correlations between the expression of the *TNNT2* fetal isoform and patient clinical data are reported in Supplementary Table 4. When considering all the muscles analyzed, the expression of the *TNNT2* fetal isoform did not correlate with patient age, while in the DM cohort, it significantly correlates with age at onset. In DM1 patients, no correlations were present between the expression of the fetal isoform and the CTG repeat expansion size while, limited to DM1 BB, the expression of *TNNT2* fetal isoform significantly correlated with MRC and MIRS.

In order to verify if the alteration of the *TNNT2* alternative splicing was more evident in DM patients who showed cardiac alterations, the levels of fetal *TNNT2* isoform were correlated to the cardiac parameters considered. Results are reported in Supplementary Table 4. Among the cardiac parameters considered, QRS showed a significant correlation with the expression of the fetal isoform of *TNNT2* in the DM1 cohort. Indeed, the correlation was more evident when considering all DM1 patients and in the DM1 CI subgroups both at the BB and TA level. On the contrary, no correlations were evident in DM2 patients. Nevertheless, it should be noted that the two DM patients with pacemaker (DM1-7 CI and DM2-9 CI) did not show the highest level of *TNNT2* fetal isoform (Figure 3A).

## Discussion

Cardiac involvement is an important cause of premature death in DM patients and one possible molecular explanation to cardiac dysfunctions is the misregulation of alternative splicing of *TNNT2* and *SCN5A* pre-mRNA in cardiac tissue (25, 26). Recently, several authors have reported that DM patients show elevated serum levels of cTnT, cTnI, and NT-pro-BNP, suggesting that these serum cardiac biomarkers could be used to identify DM patients at increased risk of developing myocardial conduction abnormalities and for stratification of subjects in clinical trials (34, 43, 46). However, to date, no cardiac biomarkers have been identified that may serve as an accurate biomarker of cardiac involvement that could be used in clinical trials.

It has been reported that in DM patients, the alternative splicing of the *TNNT2* gene, which encodes for the cardiac isoform of troponin T (cTnT), is disrupted in skeletal muscle as in cardiac muscle (25, 34, 36, 37). Thus, the aim of our study was to clarify if the expression of the *TNNT2* aberrant fetal isoform in skeletal muscle could be secondary to skeletal muscle myopathic changes or if it could reflect the cardiac phenotype, thus representing a biomarker of cardiac involvement.

We have analyzed skeletal muscle from DM1 and DM2 patients with cardiac involvement (CI) or without cardiac involvement (NCI). In DM1 patients, both proximal and distal muscles have been considered since it is known that these muscles are differently affected in this pathology, thus affecting their suitability for biomarker discovery (47). As expected, among DM1, the global histopathological alterations appear to be more compromised in the TA muscle than in the BB muscles. Moreover, patients of CI subgroups present muscles globally more compromised than those of NCI subgroups, indicating that cardiac involvement increased as the muscular disease became aggravated.

cTnT is considered a marker of skeletal muscle regeneration since, when it is injured, it repairs itself by a process that recapitulates embryonic myogenesis and causes re-expression of several fetal proteins, including cTnT and fetal myosin isoforms (31). Among all the muscles examined in our study, the highest levels of *TNNT2* total mRNA expression and the higher percentage of fibers expressing MHC-emb or MHC-neo have been observed in regenerating muscles of noDM-IBM patients. In our cohort of DM patients, a clear increase of *TNNT2* expression has been observed in DM1 TA muscles. Moreover, a significant correlation between the *TNNT2* mRNA expression levels and the percentage of MHC-neo positive fibers is present when considering either all the muscle samples analyzed or the DM cohort, and this correlation is more evident in DM1 TA muscles, confirming that this gene is more expressed in skeletal muscle expressing fetal myosins. On the contrary, no correlation is evident in DM2 patients. Nevertheless, DM skeletal muscle is not considered a regenerating tissue since it does not show the typical histological features of regenerating muscle reported in other types of muscular dystrophies. Moreover, a very low number of MHC-emb positive fibers and few MHC-neo positive fibers are observable in both DM1 (BB and TA) and DM2 muscles compared to the regenerating noDM-IBM muscles. Also, almost all fibers with central nuclei resulted negative to MHC-neo or MHC-emb immunostaining, and like many myopathies, centrally positioned nuclei in DM seem not to be linked to degeneration/regeneration processes (48). It is noteworthy that when considering the global muscle alteration using the histopathological score described in Materials and Methods, a significant correlation with cTnT expression is evident when considering all DM muscles examined or DM1 TA muscles. Taken together, these results suggest that the typical histological alterations observable in DM skeletal muscles may not be related to a degeneration/regeneration process; however, the myopathic changes in the most severely affected muscles can lead to a slight reexpression of cTnT. Nevertheless, in our western blot experiments, the antibody used detects positive bands at a molecular weight consistent with cTnT in the skeletal muscle of noDM-IBM patients and in the myocardium, but no bands have been detected in any of the DM or CTR skeletal muscles. Our data are in line with those reported by other authors on cTnT protein expression in regenerating muscles and suggest that, in DM skeletal muscle, a very low or no expression of cTnT protein is present (32, 34, 35, 49, 50). These data open the question of the biological significance of the elevated circulating cTnT found in DM patients without clinical cardiovascular disease (33, 34). Increased plasma cTnT levels in DM patients might be caused by subclinical myocardial damage not detected by conventional measures rather than to a release of cTnT protein from injured skeletal muscle into the circulation as observed in various neuromuscular diseases included IBM disease (32, 33). However, our data and recently reported data suggest that cTnT antibodies detect positive bands at a molecular weight corresponding to skeletal muscle TnT in both healthy and diseased muscle, indicating that a cross-reactivity with the skeletal muscle TnT may contribute to elevated cTnT plasma concentrations in patients with myopathies (51, 52).

It is known that *TNNT2* alternative splicing is disrupted in DM and in other neuromuscular diseases such that exon 5 is inappropriately included in adult skeletal muscle (25, 36, 45). Several studies on human patients and mouse models of muscular dystrophies report that splicing changes may be a much more general phenomenon of muscle disease and can be secondary to muscle regeneration (44, 45, 53). In order to verify this hypothesis in DM skeletal muscles, we have analyzed the DM-associated splicing defects of *TNNT2* and of several genes involved in DM skeletal muscle histological and physiological alterations. When considering the alternative splicing of muscle-specific genes, the expression of the aberrant isoforms significantly correlates with the muscle histopathological alterations. Indeed, among BB muscles, missplicing of the skeletal muscle-specific genes is more evident in DM2 patients and appears to be higher in patient groups who also present more severe muscle histopathological alterations. Moreover, the extent of splicing alterations in DM1 TA distal skeletal muscles is greater compared to that observed in DM1 BB, and it appears to be similar in the two DM1 subgroups who also show similar muscular histopathological alterations. These data confirm what was already reported by other authors in that proximal muscles are less involved than distal muscles in DM1, thus affecting their suitability for biomarker discovery (47, 54). However, despite the marked regenerative process, our IBM muscles do not express greater levels of DM-specific pathological isoforms of the muscle-specific genes than those observed in CTR, except for *RYR1*. The altered splicing of *RYR1* could explain the recent data by Amici et al. (55) who reported a lower protein expression of RyR1 in IBM muscles as compared to healthy subjects, indicating that Ca^2+^ dysregulation may contribute to muscle weakness and atrophy in this myopathy. We therefore can speculate that the splicing alterations of the skeletal muscle-specific genes observed in DM might not occur only secondary to an active remodeling process with myopathic changes but they could be effectively important in DM skeletal muscle pathology. These data are in line with those previously reported in other works (9, 13, 47, 56, 57).

When considering the *TNNT2* gene, missplicing appears to be more evident in DM1 muscles than in DM2 or noDM-IBM muscles. Indeed, despite the fact that proximal muscles are more involved in DM2 than in DM1 patients (47, 54), contrary to DM1, in DM2 BB, the *TNNT2* fetal isoform appears to be less expressed than the adult isoform. However, this evidence does not seem to be related to less severe histopathological alterations since the results of the quantitative evaluation of histopathological parameters appear to be similar in DM1 BB and DM2 BB muscles. Also, in regenerating muscle of noDM-IBM patients, a weak increase of the fetal isoform expression is present compared to CTR; however, the adult isoform is the prevalent isoform expressed in these muscles. These data seem to indicate that the more severe *TNNT2* missplicing observed in DM1 compared to the other muscles analyzed could not be related only to myopathic changes but could reflect the more severe general phenotype compared to DM2, including cardiac problems that appear to be more severe and frequent in patients with DM1 than in patients with DM2 (19). In order to verify if the more severe *TNNT2* missplicing observed in DM1 muscle could reflect a more severe cardiac involvement, the expression of *TNNT2* fetal isoform has been correlated to cardiac parameters obtained by ECG, ECG-Holter, or echocardiogram. The results of this analysis show that when considering all DM patients, *TNNT2* missplicing significantly correlates with the QRS cardiac parameter, and this correlation is evident in DM1 but not in DM2 patients. Of interest, the significant correlation is present also when considering the DM1-CI subgroups. This result is of particular interest since it has been reported that the gradual prolongation of QRS duration in patients affected by DM suggests the contribution of progressive fibrosis to conduction delay in this disease (58). Taken together, these results seem to indicate that the expression levels of the *TNNT2* fetal isoform could reflect the general phenotype including the cardiac involvement of DM1 but not of DM2 patients. It is known that different pathomolecular mechanisms may contribute to the lesser severity of DM2 compared to DM1. Recently, it has been reported that the rbFOX proteins may participate to the lesser toxicity of the CCTG repeat expansion in DM2 since they compete with and reduce the titration of MBNL1 within CCUG but not within CUG RNA foci (59).

The results of our study do not allow us to conclude that *TNNT2* missplicing may be considered a specific cardiac biomarker in adult skeletal muscle of DM1 patients and further investigations will be necessary to support this hypothesis. However, our data suggest that this splicing event has good potential to function as a biomarker of DM1 severity, and it should be considered in pharmacological clinical trials to monitor the possible effects of different therapeutic approaches on muscle tissues.

There are two important limitations to be acknowledged in the present study. The first limitation is the study cohort's limited sample size in some subgroups that is due to the rareness of these pathologies; thus, our evidences may not be conclusive. A second limitation regards the cardiac data taken into account. Indeed, only echocardiographic data on myocardial structure have been considered in the study, and probably, cardiac MRI data could be useful to identify the presence of myocardial morphological alterations since it is a more accurate and highly reproducible technique for assessment of cardiac volumes, function, mass, and fibrosis.

## Data Availability Statement

All datasets generated for this study are included in the manuscript/Supplementary Files.

## Ethics Statement

The studies involving human participants were reviewed and approved by Ospedale San Raffaele (Milan, Italy). The patients/participants provided their written informed consent to participate in this study.

## Author Contributions

FB performed conceptualization of the study, performed all the biomolecular analysis and the relative interpretation of data, and participated in manuscript drafting and final revision. LR performed western blotting and the relative interpretation of data and participated in manuscript final revision. BF and GA performed neurological examination and revision of the manuscript. VL performed the collection and critical analysis of the cardiac data and revision of the manuscript. VM made substantial contributions to the statistical analysis and the interpretation of the data and revision of the manuscript. AB performed critical analysis of the results and performed critical revision of manuscript. EM performed critical revision of manuscript. GM performed neurological examination and performed critical revision of manuscript. RC performed conceptualization of the study, carried out skeletal muscle biopsies, performed muscle histopathological evaluation, data analysis and interpretation, participated in manuscript drafting, and gave the final approval of the manuscript.

### Conflict of Interest

The authors declare that the research was conducted in the absence of any commercial or financial relationships that could be construed as a potential conflict of interest.

## References

[1] Harper PS. Myotonic Dystrophy. 3rd edn. London: WB Saunders (2001).

[2] Brook JD McCurrach ME Harley HG Buckler AJ Church D Aburatani H. Molecular basis of myotonic dystrophy: Expansion of a trinucleotide (CTG) repeat at the 3′end of a transcript encoding a proteinkinase family member. Cell. (1992) 69:799–808. 10.1016/0092-8674(92)90154-5 1310900

[3] Fu YH Pizzuti A Fenwick RG Jr King J Rjnarayan S Dunne PW. An unstable triplet repeat in a gene related to myotonic muscular dystrophy. Science. (1992) 255:1256–8. 10.1126/science.1546326 1546326

[4] Mahadevan M Tsilfidis C Sabourin L Shutler G Amemiya C Jansen G. Myotonic dystrophy mutation: an unstable CTG repeat in the 3′ untranslated region of the gene. Science. (1992) 255:1253–5. 10.1126/science.1546325 1546325

[5] Liquori CL Ricker K Moseley ML Jacobsen JF Kress W Naylor SL. Myotonic dystrophy type 2 caused by a CCTG expansion in intron 1 of ZNF9. Science. (2001) 293:864–7. 10.1126/science.1062125 11486088

[6] Sznajder ŁJ Swanson MS. Short tandem repeat expansions and RNA-mediated pathogenesis in myotonic dystrophyInt. J Mol Sci. (2019) 20:E3365. 10.3390/ijms20133365 31323950 PMC6651174

[7] Savkur RS Philips AV Cooper TA. Aberrant regulation of insulin receptor alternative splicing is associated with insulin resistance in myotonic dystrophy. Nat Genet. (2001) 29:40–7. 10.1038/ng704 11528389

[8] Savkur RS Philips AV Cooper TA Dalton JC Moseley ML Ranum LP. Insulin receptor splicing alteration in myotonic dystrophy type 2. Am J Hum Genet. (2004) 74:1309–13. 10.1086/421528 15114529 PMC1182097

[9] Mankodi A Takahashi MP Jiang H Beck CL Bowers WJ Moxley RT. Expanded CUG repeats trigger aberrant splicing of ClC-1chloride channel pre-mRNA and hyperexcitability of skeletal muscle in myotonic dystrophy. Mol Cell. (2002) 10:35–44. 10.1016/S1097-2765(02)00563-4 12150905

[10] Charlet-B N Savkur RS Singh G Philips AV Grice EA Cooper TA. Loss of the muscle-specific chloride channel in type 1 myotonic dystrophy due to misregulated alternative splicing. Mol Cell. (2002) 10:45–53. 10.1016/S1097-2765(02)00572-5 12150906

[11] Wheeler TM Lueck JD Swanson MS Dirksen RT Thornton CA. Correction of ClC-1 splicing eliminates chloride channelopathy and myotonia in mouse models of myotonic dystrophy. J Clin Invest. (2007) 117:3952–7. 10.1172/JCI33355 18008009 PMC2075481

[12] Rau F Lainé J Ramanoudjame L Ferry A Arandel L Delalande O. Abnormal splicing switch of DMD's penultimate exon compromises muscle fibre maintenance in myotonic dystrophy. Nat Commun. (2015) 6:7205. 10.1038/ncomms8205 26018658 PMC4458869

[13] Kimura T Nakamori M Lueck JD Pouliquin P Aoike F Fujimura H. Altered mRNA splicing of the skeletal muscle ryanodine receptor and sarcoplasmic/endoplasmic reticulum Ca2þ-ATPase in myotonic dystrophy type 1. Hum Mol Genet. (2005) 14:2189–00. 10.1093/hmg/ddi223 15972723

[14] Fugier C Klein AF Hammer C Vassilopoulos S Ivarsson Y Toussaint A. Misregulated alternative splicing of BIN1 is associated with T tubule alterations and muscle weakness in myotonic dystrophy. Nat Med. (2011) 17:720–5. 10.1038/nm.2374 21623381

[15] Tang ZZ Yarotskyy V Wei L Sobczak K Nakamori M Eichinger K. Muscle weakness in myotonic dystrophy associated with misregulated splicing and altered gating of Ca(V)1.1 calcium channel. Hum Mol Genet. (2012) 21:1312–24. 10.1093/hmg/ddr568 22140091 PMC3284119

[16] Botta A Malena A Loro E Del Moro G Suman M Pantic B. Altered Ca2+ homeostasis and endoplasmic reticulum stress in myotonic dystrophy type 1 muscle cells. Genes. (2013) 4:275–92. 10.3390/genes4020275 24705164 PMC3899969

[17] Groh WJ Groh MR Saha C Kincaid JC Simmons Z Ciafaloni E. Electrocardiographic abnormalities and sudden death in myotonic dystrophy type 1. N Engl J Med. (2008) 358:2688–97. 10.1056/NEJMoa062800 18565861

[18] Lau JK Sy RW Corbett A Kritharides L. Myotonic dystrophy and the heart: a systematic review of evaluation and management. Int J Cardiol. (2015) 184:600–8. 10.1016/j.ijcard.2015.03.069 25769007

[19] Sansone VA Brigonzi E Schoser B Villani S Gaeta M De Ambroggi G. The frequency and severity of cardiac involvement in myotonic dystrophy type 2 (DM2): long-term outcomes. Int J Cardiol. (2013) 168:1147–53. 10.1016/j.ijcard.2012.11.076 23266299

[20] Wahbi K Meune C Bécane HM Laforêt P Bassez G Lazarus A. Left ventricular dysfunction and cardiac arrhythmias are frequent in type 2 myotonic dystrophy: a case control study. Neuromuscul Disord. (2009) 19:468–72. 10.1016/j.nmd.2009.04.012 19481939

[21] Petri H Vissing J Witting N Bundgaard H Køber L. Cardiac manifestations of myotonic dystrophy type 1. Int J Cardiol. (2012) 160:82–8. 10.1016/j.ijcard.2011.08.037 21917328

[22] Wahbi K Meune C Porcher R Bécane HM Lazarus A Laforêt P. Electrophysiological study with prophylactic pacing and survival in adults with myotonic dystrophy and conduction system disease. JAMA. (2012) 307:1292–301. 10.1001/jama.2012.346 22453570

[23] Wahbi K Babuty D Probst V Wissocque L Labombarda F Porcher R. Incidence and predictors of sudden death, major conduction defects and sustained ventricular tachyarrhythmias in 1388 patients with myotonic dystrophy type 1. Eur Heart J. (2017) 38:751–8. 10.1093/eurheartj/ehw569 27941019

[24] Wahbi K Porcher R Laforêt P Fayssoil A Bécane HM Lazarus A. Development and validation of a new scoring system to predict survival in patients with myotonic dystrophy type 1. JAMA Neurol. (2018) 7:573–81. 10.1001/jamaneurol.2017.4778 PMC5885178 29404559

[25] Philips AV Timchenko LT Cooper TA. Disruption of splicing regulated by a CUG-binding protein in myotonic dystrophy. Science. (1998) 280:737–41. 10.1126/science.280.5364.737 9563950

[26] Freyermuth F Rau F Kokunai Y Linke T Sellier C Nakamori M. Splicing misregulation of SCN5A contributes to cardiac conduction delay and heart arrhythmia in myotonic dystrophy. Nat Commun. (2016) 7:11067. 10.1038/ncomms11067 27063795 PMC4831019

[27] Anderson PA Malouf NN Oakeley AE Pagani ED Allen PD. Troponin T isoform expression in humans. A comparison among normal and failing adult heart, fetal heart, and adult and fetal skeletal muscle. Circ Res. (1991) 69:1226–33. 10.1161/01.RES.69.5.1226 1934353

[28] Anderson PA Greig A Mark TM Malouf NN Oakeley AE Ungerleider RM. Molecular basis of human cardiac troponin T isoforms expressed in the developing, adult, and failing heart. Circ Res. (1995) 76:681–6. 10.1161/01.RES.76.4.681 7534662

[29] Godt RE Fogaca RTH Silva IK Nosek TM. Contraction of developing avian heart muscle. Comp Biochem Physiol. (1993) 105:213–8. 10.1016/0300-9629(93)90197-C 8101153

[30] McAuliffe JJ Gao LZ Solaro RJ. Changes in myofibrillar activation and troponin C Ca2+ binding associated with troponin T isoform switching in developing rabbit heart. Circ Res. (1990) 66:1204–16. 10.1161/01.RES.66.5.1204 2139820

[31] Ciciliot S Schiaffino S. Regeneration of mammalian skeletal muscle. Basic mechanisms and clinical implications. Curr Pharm Des. (2010) 16:906–14. 10.2174/138161210790883453 20041823

[32] Jaffe AS Vasile VC Milone M Saenger AK Olson KN Apple FS. Diseased skeletal muscle: a noncardiac source of increased circulating concentrations of cardiac troponin T. J Am Coll Cardiol. (2011) 58:1819–24. 10.1016/j.jacc.2011.08.026 21962825

[33] Rittoo D Jones A Lecky B Neithercut D. Elevation of cardiac troponin T, but not cardiac troponin I, in patients with neuromuscular diseases: implications for the diagnosis of myocardial infarction. J Am Coll Cardiol. (2014) 63:2411–20. 10.1016/j.jacc.2014.03.027 24747102

[34] Valaperta R Gaeta M Cardani R Lombardi F Rampoldi B De Siena C. High-sensitive cardiac troponin T (hs-cTnT) assay as serum biomarker to predict cardiac risk in myotonic dystrophy: a case–control study. Clin Chim Acta. (2016) 463:122–8. 10.1016/j.cca.2016.10.026 27780717

[35] Wens SC Schaaf GJ Michels M Kruijshaar ME van Gestel TJ In't Groen S. Elevated plasma cardiac troponin T levels caused by skeletal muscle damage in Pompe disease. Circ Cardiovasc Genet. (2016) 9:6–13. 10.1161/CIRCGENETICS.115.001322 26787432

[36] Botta A Rinaldi F Catalli C Vergani L Bonifazi E Romeo V. The CTG repeat expansion size correlates with the splicing defects observed in muscles from myotonic dystrophy type 1 patients. J Med Genet. (2008) 45:639–46. 10.1136/jmg.2008.058909 18611984

[37] Santoro M Masciullo M Bonvissuto D Bianchi ML Michetti F Silvestri G. Alternative splicing of human insulin receptor gene (INSR) in type I and type II skeletal muscle fibers of patients with myotonic dystrophy type 1 and type 2. Mol Cell Biochem. (2013) 380:259–65. 10.1007/s11010-013-1681-z 23666741

[38] Moxley RT Meola G Udd B Ricker K. Report of the 84th ENMC workshop: PROMM (proximal myotonic myopathy) and other myotonic dystrophy-like syndromes: 2nd workshop. 13-15th October 2000. Loosdrecht, The Netherlands. Neuromuscul Disord. (2002) 12:306–17. 10.1016/S0960-8966(01)00284-X 11801405

[39] Cardani R Mancinelli E Sansone V Rotondo G Meola G. Biomolecular identification of (CCTG)n mutation in myotonic dystrophy type 2 (DM2) by FISH on muscle biopsy. Eur J Histochem. (2004) 48:437–42. 10.4081/918 15718211

[40] Valaperta R Sansone V Lombardi F Verdelli C Colombo A Valisi M. Identification and characterization of DM1 patients by a new diagnostic certified assay: neuromuscular and cardiac assessments. Biomed Res Int. (2013) 2013:958510. 10.1155/2013/958510 23762868 PMC3665172

[41] Mathieu J Boivin H Meunier D Gaudreault M Begin P. Assessment of a disease-specific muscular impairment rating scale in myotonic dystrophy. Neurology. (2001) 56:336–40. 10.1212/WNL.56.3.336 11171898

[42] Vihola A Bassez G Meola G Zhang S Haapasalo H Paetau A. Histopathological differences of myotonic dystrophy type 1 (DM1) and PROMM/DM2. Neurology. (2003) 60:1854–7. 10.1212/01.WNL.0000065898.61358.09 12796551

[43] Valaperta R De Siena C Cardani R Lombardia F Cenko E Rampoldi B. Cardiac involvement in myotonic dystrophy: the role of troponins and N-terminal pro B-type natriuretic peptide. Atherosclerosis. (2017) 267:110–5. 10.1016/j.atherosclerosis.2017.10.020 29121498

[44] Orengo JP Ward AJ Cooper TA. Alternative splicing dysregulation secondary to skeletal muscle regeneration. Ann Neurol. (2011) 69:681–90. 10.1002/ana.22278 21400563 PMC3082633

[45] Bachinski LL Baggerly KA Neubauer VL Nixon TJ Raheem O Sirito M. Most expression and splicing changes in myotonic dystrophy type 1 and type 2 skeletal muscle are shared with other muscular dystrophies. Neuromuscul Disord. (2014) 24:227–40. 10.1016/j.nmd.2013.11.001 24332166 PMC3943873

[46] Hamilton MJ Robb Y Cumming S Gregory H Duncan A Rahman M. Elevated plasma levels of cardiac troponin-I predict left ventricular systolic dysfunction in patients with myotonic dystrophy type 1: a multicentre cohort follow-up study. PLoS ONE. (2017) 12:e0174166. 10.1371/journal.pone.0174166 28323905 PMC5360313

[47] Nakamori M Sobczak K Puwanant A Welle S Eichinger K Pandya S. Splicing biomarkers of disease severity in myotonic dystrophy. Ann Neurol. (2013) 74:862–72. 10.1002/ana.23992 23929620 PMC4099006

[48] Roman W Gomes ER. Nuclear positioning in skeletal muscle. Semin Cell Dev Biol. (2018) 82:51–6. 10.1016/j.semcdb.2017.11.005 29241690

[49] Bodor GS Survant L Voss EM Smith S Porterfield D Apple FS. Cardiac troponin T composition in normal and regenerating human skeletal muscle. Clin Chem. (1997) 43:476–84. 9068591

[50] Ricchiuti V Voss EM Ney A Odland M Anderson PA Apple FS. Cardiac troponin T isoforms expressed in renal diseased skeletal muscle will not cause false-positive results by the second generation cardiac troponin T assay by Boehringer Mannheim. Clin Chem. (1998) 44:1919–24. 9732977

[51] Schmid J Liesinger L Birner-Gruenberger R Stojakovic T Scharnagl H Dieplinger B. Elevated cardiac troponin T in patients with skeletal myopathies. J Am Coll Cardiol. (2018) 71:1540–9. 10.1016/j.jacc.2018.01.070 29622161

[52] Vroemen WHM de Boer D Streng AS Mingels AMA Meex SJR. Elevated cardiac troponin T in skeletal myopathies: Skeletal TnT cross-reactivity and/or cardiac TnT expression? J Am Coll Cardiol. (2018) 72:347–9. 10.1016/j.jacc.2018.05.017 30012331

[53] Bachinski LL Sirito M Bohme M Baggerly KA Udd B Krahe R. Altered MEF2 isoforms in myotonic dystrophy and other neuromuscular disorders. Muscle Nerve. (2010) 42:856–63. 10.1002/mus.21789 21104860 PMC4136472

[54] Cardani R Giagnacovo M Rossi G Renna LV Bugiardini E Pizzamiglio C. Progression of muscle histopathology but not of spliceopathy in myotonic dystrophy type 2. Neuromuscul Disord. (2014) 24:1042–53. 10.1016/j.nmd.2014.06.435 25139674

[55] Amici DR Pinal-Fernandez I Mázala DA Lloyd TE Corse AM Christopher-Stine L. Calcium dysregulation, functional calpainopathy, and endoplasmic reticulum stress in sporadic inclusion body myositis. Acta Neuropathol Commun. (2017) 5:24. 10.1186/s40478-017-0427-7 28330496 PMC5363023

[56] Nakamori M Kimura T Fujimura H Takahashi MP Sakoda S. Altered mRNA splicing of dystrophin in type 1 myotonic dystrophy. Muscle Nerve. (2007) 36:251–7. 10.1002/mus.20809 17487865

[57] Perfetti A Greco S Fasanaro P Bugiardini E Cardani R Garcia-Manteiga JM. Genome wide identification of aberrant alternative splicing events in myotonic dystrophy type 2. PLoS ONE. (2014) 9:e93983. 10.1371/journal.pone.0093983 24722564 PMC3983107

[58] Nazarian S Bluemke DA Wagner KR Zviman MM Turkbey E Caffo BS. QRS prolongation in myotonic muscular dystrophy and diffuse fibrosis on cardiac magnetic resonance. Magn Reson Med. (2010) 64:107–14. 10.1002/mrm.22417 20572151 PMC3034129

[59] Sellier C Cerro-Herreros E Blatter M Freyermuth F Gaucherot A Ruffenach F. rbFOX1/MBNL1 competition for CCUG RNA repeats binding contributes to myotonic dystrophy type 1/type 2 differences. Nat Commun. (2018) 9:2009. 10.1038/s41467-018-04370-x 29789616 PMC5964235

